# An In Vitro Comparative Study of the Effects of Tetrabromobisphenol A and Tetrabromobisphenol S on Human Erythrocyte Membranes—Changes in ATP Level, Perturbations in Membrane Fluidity, Alterations in Conformational State and Damage to Proteins

**DOI:** 10.3390/ijms22179443

**Published:** 2021-08-31

**Authors:** Monika Jarosiewicz, Piotr Duchnowicz, Paweł Jarosiewicz, Bogumiła Huras, Bożena Bukowska

**Affiliations:** 1 Department of Biophysics of Environmental Pollution, Faculty of Biology and Environmental Protection, University of Lodz, Pomorska 141/143, 90-236 Lodz, Poland; piotr.duchnowicz@biol.uni.lodz.pl (P.D.); bozena.bukowska@biol.uni.lodz.pl (B.B.); 2 Department of Cytobiochemistry, Faculty of Biology and Environmental Protection, University of Lodz, Pomorska 141/143, 90-236 Lodz, Poland; 3 European Regional Centre for Ecohydrology of the Polish Academy of Sciences, Tylna 3, 90-364 Lodz, Poland; p.jarosiewicz@erce.unesco.lodz.pl; 4 Łukasiewicz Research Network, Institute of Industrial Organic Chemistry, Annopol 6 Str, 03-236 Warsaw, Poland; bogumila.huras@ipo.lukasiewicz.gov.pl

**Keywords:** tetrabromobisphenol A, tetrabromobisphenol S, erythrocyte membrane, retardants, erythrocytes

## Abstract

Brominated flame retardants (BFRs) are substances used to reduce the flammability of plastics. Among this group, tetrabormobisphenol A (TBBPA) is currently produced and used on the greatest scale, but due to the emerging reports on its potential toxicity, tetrabromobisphenol S (TBBPS)—a compound with a very similar structure—is used as an alternative. Due to the fact that the compounds in question are found in the environment and in biological samples from living organisms, including humans, and due to the insufficient toxicological knowledge about them, it is necessary to assess their impacts on living organisms and verify the validity of TBBPA replacement by TBBPS. The RBC membrane was chosen as the research model. This is a widely accepted research model for assessing the toxicity of xenobiotics, and it is the first barrier to compounds entering circulation. It was found that TBBPA and TBBPS caused increases in the fluidity of the erythrocyte membrane in their hydrophilic layer, and conformational changes to membrane proteins. They also caused thiol group elevation, an increase in lipid peroxidation (TBBPS only) and decreases in the level of ATP in cells. They also caused changes in the size and shape of RBCs. TBBPA caused changes in the erythrocyte membrane at lower concentrations compared to TBBPS at an occupational exposure level.

## 1. Introduction

Tetrabromobisphenol A (TBBPA) is a compound belonging to the group of brominated flame retardants (BFRs). These compounds have been used since the 1970s to reduce the flammability of plastics in many consumer products, such as household articles, furniture, mattresses (including products for babies), textiles, insulation and electronic equipment housings [1]. Currently, TBBPA, produced in the amount of over 200 thousand tons per year, is the most important among that group of compounds [2]. It is worth noting that the production of TBBPA accounts for approximately 60% of all BFRs used, and it is not subject to monitoring or restrictions. This is largely due to the fact that most TBBPA (approximately 90%) is used as a reactive compound, i.e., covalently bonded to a polymer matrix, which limits the possibility of its migration to the environment, but the rest is used in an additive form, which can be released from the product much more easily [3].

TBBPA’s widespread use has contributed to the contamination of the environment. TBBPA was found in environmental samples such as soil, water and air [4,5,6,7]. It was also found in the air and dust of residential interiors and offices, which significantly contributes to human exposure [8,9]. TBBPA, due to its wide presence in the natural environment and direct human environment, may pose a significant risk to health. Due to the high hydrophobicity of the compound, it can bioaccumulate in living organisms, including humans [10]. Numerous studies document the presence of TBBPA in adipose tissue, milk and serum of mothers, and also in neonatal serum, which is particularly disturbing [10,11]. Currently, more and more publications are appearing indicating the potential toxicity of high doses of TBBPA for mammals. It was found that this compound may be involved in the development of many diseases, such as diabetes, or participate in the neoplastic process [12,13,14].

Tetrabromobisphenol S (TBBPS) is increasingly being used as an alternative to TBBPA. These compounds have very similar molecular structures, with one difference: in TBBPS, sulfone groups are present instead of the methyl groups of TBBPA. The use of this compound is supported by the presence of a sulfone group in the molecule, which may limit its toxicity, and the fact that this compound is characterized by better flame retardancy [15]. Additionally, apart from the fact that TBBPS is used as a flame retardant additive, it is also used as a herbicide, to control selected weeds in the cultivation of cucumbers, tomatoes and white radish [16]. The increasing use of this compound and its derivatives is resulting in greater presence in the ecosystem and the exposure of living organisms to it. The compound was found, among others, in aquatic organisms and blood serum samples from pregnant women in China [17,18]. So far, there are few toxicological studies that could answer the question of the safety of TBBPS as an alternative. However, due to the structural similarity of the two compounds discussed, it seems reasonable to assume that they may have similar potentially adverse effects on living organisms. Therefore, it is necessary to determine and compare the toxicities of TBBPA and TBBPS.

The erythrocyte membrane is a widely accepted research model in the assessment of xenobiotic toxicity. The changes within it correlate with changes in the membranes of other cell types. Damage to the cell membrane may affect its function and contribute to cell death [19]. Changes in the properties of cell membrane may also be involved in the development of many diseases, including anemia, diabetes, heart diseases and cancer. It has been found that TBBPA may contribute to the induction of cancer and may be involved in the development of type II diabetes and obesity [12,13,14], therefore, the mechanisms of action of TBBPA and TBBPS in the model cell membrane should be investigated. Therefore, the aim of the study was to evaluate the influences of common BFRs, i.e., TBBPA and TBBPS, on the properties of the erythrocyte membrane.

## 2. Results

### 2.1. Membrane Fluidity

In RBCs, an increase in fluidity of the hydrophilic layer of the membrane was observed (an increase in the order parameter S). No statistically significant changes were observed in fluidity of deeper regions of the lipid bilayer.

Both compounds caused an increase in the value of the S parameter proportional to the concentration (5-DSA labeling). There were slight statistically significant differences with the lowest tested concentration of TBBPA (1 µg/mL) and with 10 µg/mL of TBBPS. Changes in relation to the control, both of approximately 105%, were recorded for TBBPA at the concentration of 25 µg/mL, and for TBBPS at twice that high concentration. However, no significant changes were observed in the correlation time coefficients τB and τC (16-DSA labeling) (Table 1).

### 2.2. W/S Ratio

**Figure 1:**
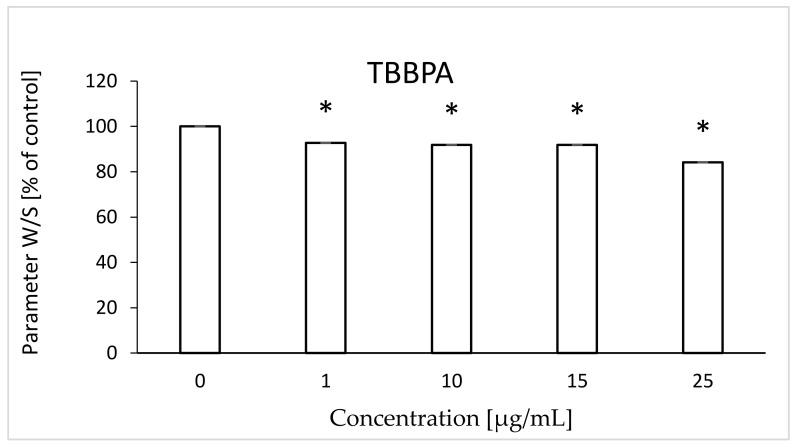
Changes in W/S in human control erythrocytes and the erythrocytes incubated with TBBPA at 1 to 25 μg/mL for 48 h. (*) Significantly different from control (*p* < 0.05).

**Figure 2:**
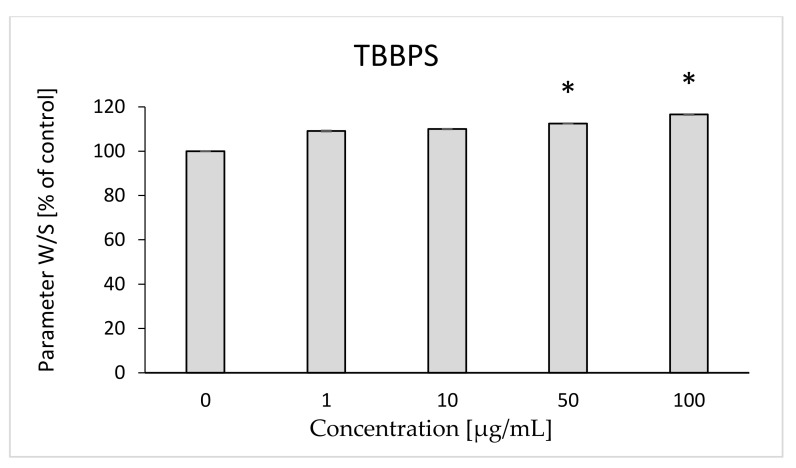
Changes in W/S in human control erythrocytes and the erythrocytes incubated with TBBPS at 1 to 100 μg/mL for 48 h. (*) Significantly different from control (*p* < 0.05).

It was found that TBBPA caused a statistically significant decrease in mobility of the attached marker at the lowest concentration; changes ranged from 92.7 to 84.1% (for 1 µg/mL and 25 µg/mL, respectively) compared to the control (100%) (Figure 1). On the other hand, TBBPS caused statistically significant increases in the mobility of the attached marker at the concentrations of 50 and 100 µg/mL (112.52 and 116.53%, respectively) (Figure 2).

### 2.3. Internal Viscosity of Erythrocytes

It was observed that the compounds slightly increased the intrinsic viscosity of RBCs, but these changes were statistically insignificant (Table 2).

### 2.4. Thiol Groups

**Figure 3:**
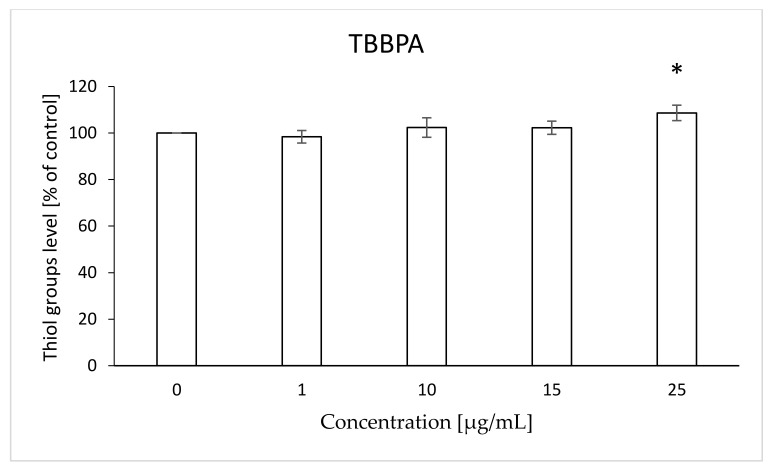
Changes in the thiol groups level in human control erythrocytes and the erythrocytes incubated with TBBPA at 1 to 25 μg/mL for 12 h. (*) Significantly different from control (*p* < 0.05).

**Figure 4:**
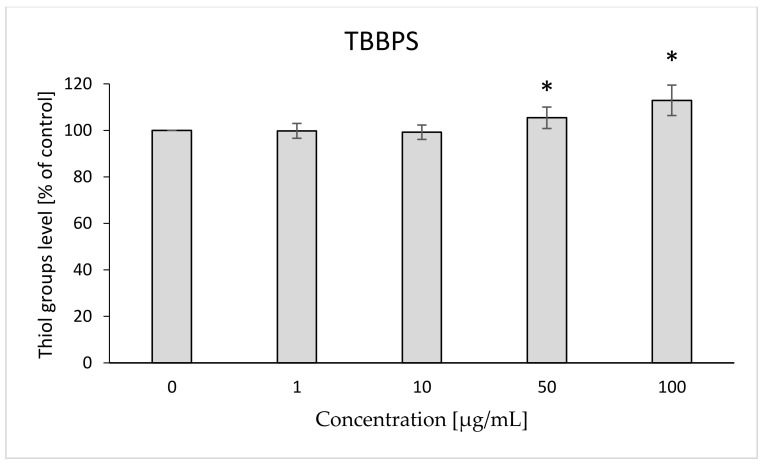
Changes in the thiol groups level in human control erythrocytes and the erythrocytes incubated with TBBPS at 1 to 100 μg/mL for 12 h. (*) Significantly different from control (*p* < 0.05).

The level of thiol groups in erythrocyte membrane was assessed after 12 h of incubation with the analyzed compounds. It was found that both TBBPA and TBBPS caused statistically significant increases in that parameter. TBBPA at the concentration of 25 µg/mL increased the level of thiol groups by 8% compared to the control (Figure 3). Statistically significant changes for TBBPS were observed at concentrations of 50 and 100 µg/mL (by 5 and 12% compared to the control, respectively) (Figure 4).

### 2.5. ATP Level

**Figure 5:**
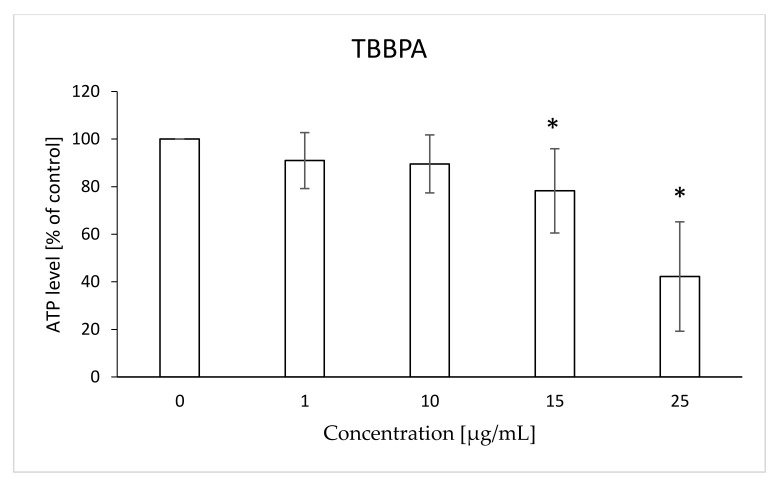
Changes in ATP level in human control erythrocytes and the erythrocytes incubated with TBBPA at 1 to 25 μg/mL for 12 h. (*) Significantly different from control (*p* < 0.05).

**Figure 6:**
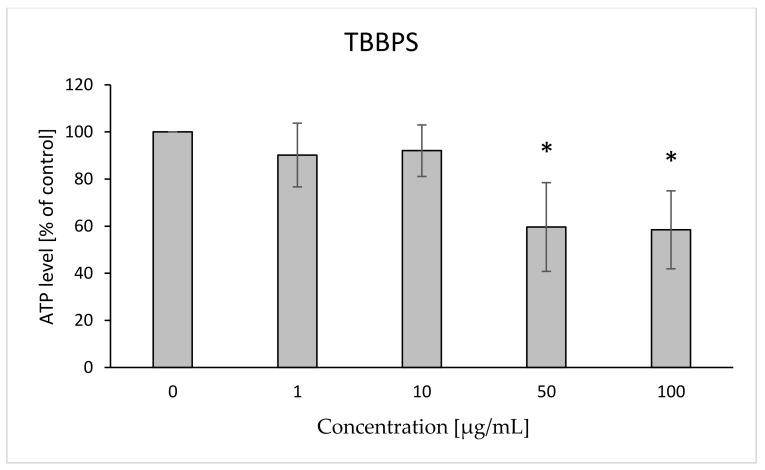
Changes in ATP level in human control erythrocytes and the erythrocytes incubated with TBBPS at 1 to 100 μg/mL for 12 h. (*) Significantly different from control (*p* < 0.05).

It was found that the tested bromobisphenols reduced the level of intracellular ATP. TBBPA caused statistically significant decreases in ATP level compared to the control at concentrations of 15 and 25 µg/mL—78 and 42%, respectively (Figure 5). In the case of TBBPS, statistically significant decreases in the discussed parameter in relation to the control were observed at the concentrations of 50 and 100 µg/mL—60 and 58%, respectively (Figure 6).

### 2.6. Lipid Peroxidation

**Figure 7:**
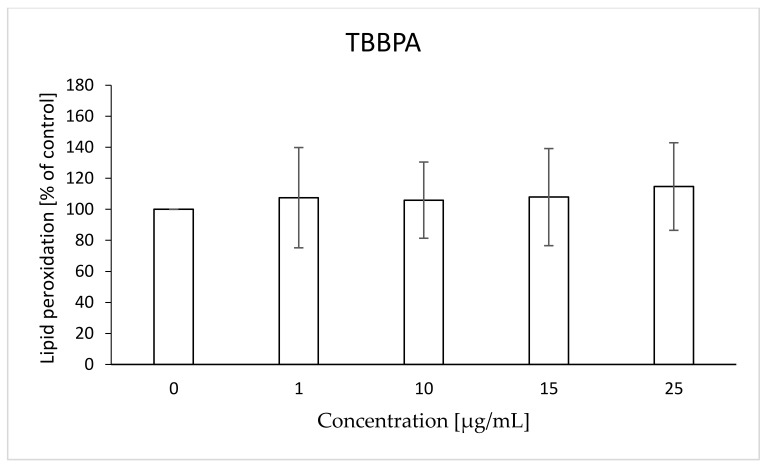
Lipid peroxidation in human control erythrocytes and the erythrocytes incubated with TBBPA at 1 to 25 μg/mL for 48 h.

**Figure 8:**
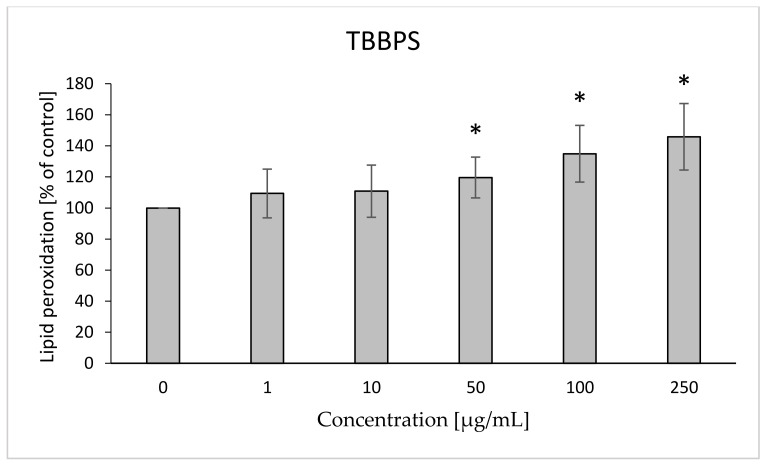
Lipid peroxidation in human control erythrocytes and the erythrocytes incubated with TBBPS at 1 to 250 μg/mL for 48 h. (*) Significantly different from control (*p* < 0.05).

After 48 h of incubation of RBCs with the analyzed compoundsin the case of TBBPA, no statistically significant changes were found (Figure 7) within the range of pre-hemolytic concentrations. Statistically significant increases in lipid peroxidation under the influence of TBBPS were observed for concentrations of 50, 100 and 250 µg/mL (up to 120, 135, 145%, respectively) (Figure 8).

### 2.7. Osmotic Fragility

**Figure 9:**
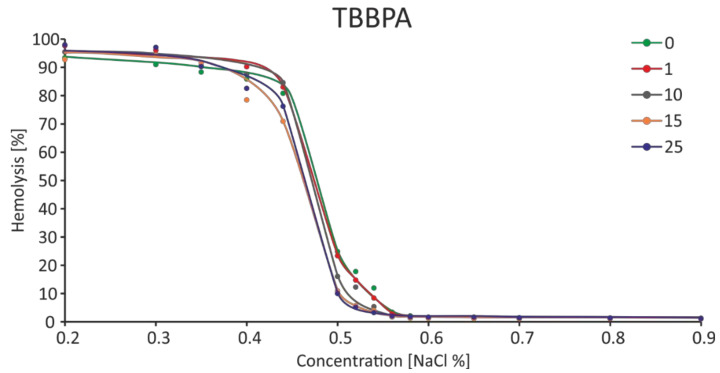
Changes in osmotic resistance of human erythrocytes incubated with TBBPA at 1 to 25 μg/mL for 3 h.

**Figure 10:**
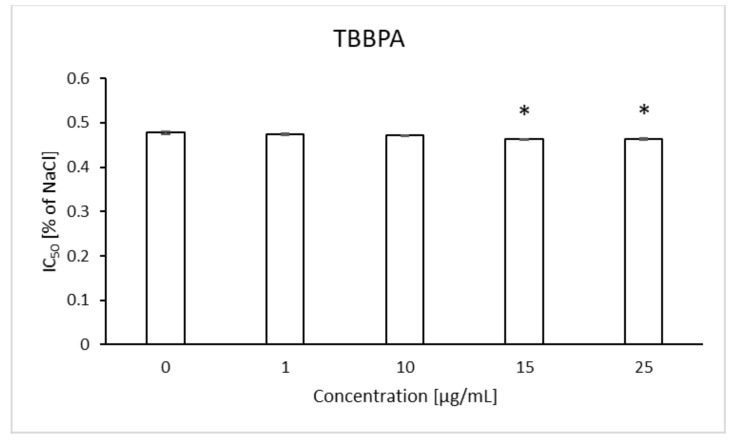
IC_50_ parameter for control erythrocytes and erythroctes incubated with TBBPA at 1–25 μg/mL for 3 h. (*) Significantly different from control (*p* < 0.05).

**Figure 11:**
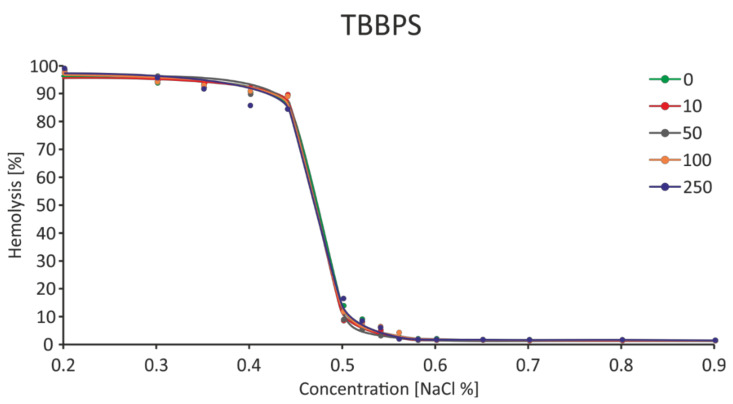
Changes in osmotic resistance of human erythrocytes incubated with TBBPS at 1 to 250 μg/mL for 3 h.

**Figure 12:**
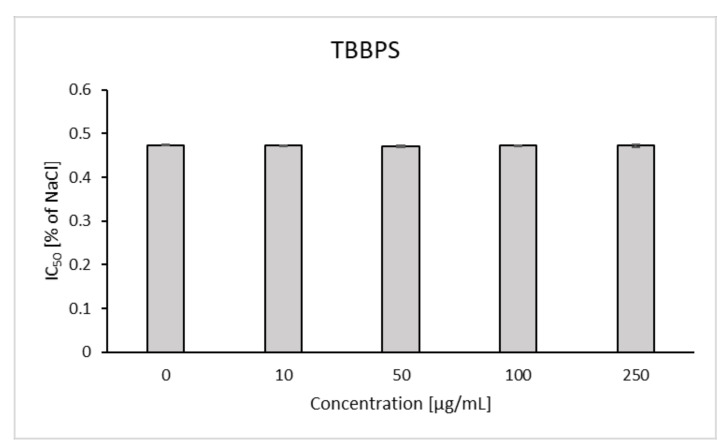
IC_50_ parameter for control erythrocytes and erythroctes incubated with TBBPS at 10–250 μg/mL for 3 h.

It was observed that TBBPA at the lowest concentration (1 µg/mL) caused a slight increase in osmotic resistance, and the highest increases of this parameter were found for the concentration of 15 and 25 µg/mL. At the concentration of NaCl equal to 0.52%, decreases in RBC hemolysis were found to be 6.09 and 5.57%, with the control value of 18.18% (Figure 9). It was shown that in the presence of TBBPA there was a statistically significant decrease in the IC_50_ value for NaCl (Figure 10). On the other hand, there was no effect of TBBPS on the RBC osmotic resistance under the influence of various concentrations of NaCl, nor were there changes in the IC_50_ value (Figure 11 and Figure 12).

### 2.8. Morphological Changes of Erythrocytes, FSC and SSC Parameter

**Figure 13:**
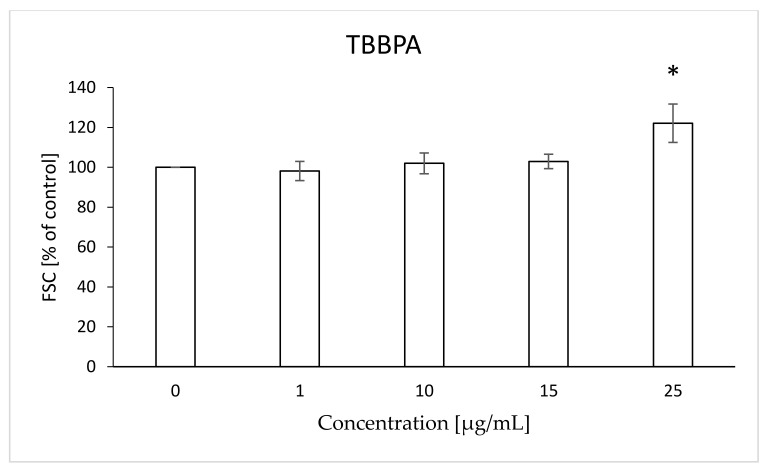
Changes in the FSC parameter in human control erythrocytes and the erythrocytes incubated with TBBPA at 1 to 25 μg/mL for 48 h. (*) Significantly different from control (*p* < 0.05).

**Figure 14:**
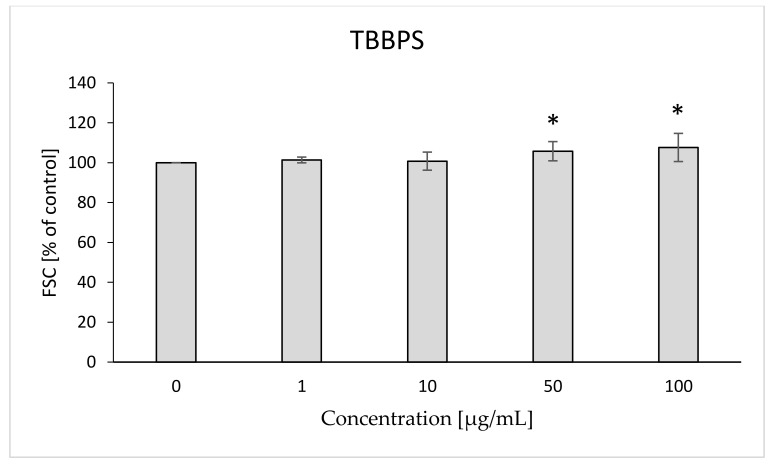
Changes in the FSC parameter in human control erythrocytes and the erythrocytes incubated with TBBPS at 1 to 100 μg/mL for 48 h. (*) Significantly different from control (*p* < 0.05).

The analyzed compounds after 48 h of incubation with RBCs caused changes in the FSC and SSC parameters that can be used to assess the size and shape of the cell. After 48 h of RBC incubation with bromobisphenols both compounds increased the FSC parameter, compared to control erythrocytes. There was a statistically significant increase in FSC caused by TBBPA at 25 µg/mL (122%) (Figure 13) and TBBPS at 50 and 100 µg/mL (106, 108%) (Figure 14).

**Figure 15:**
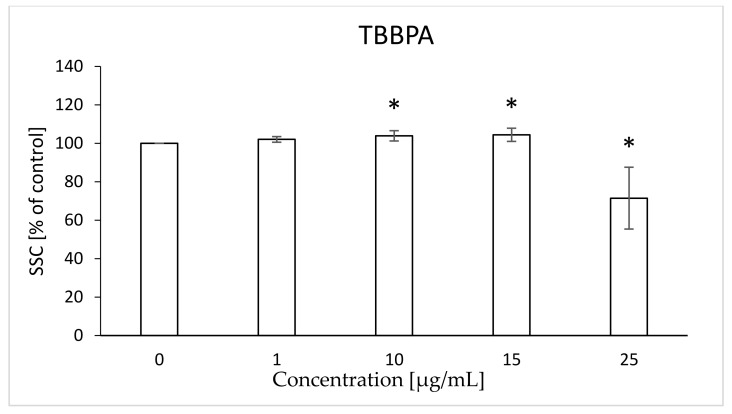
Changes in the SSC parameter in human control erythrocytes and the erythrocytes incubated with TBBPA at 1 to 25 μg/mL for 48 h. (*) Significantly different from control (*p* < 0.05).

**Figure 16:**
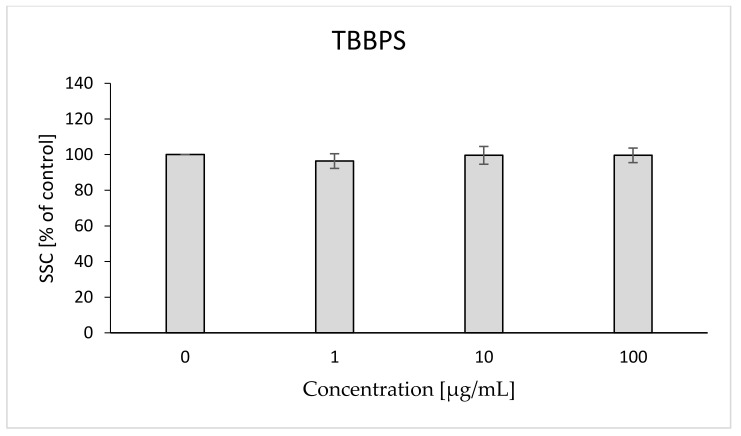
Changes in the SSC parameter in human control erythrocytes and the erythrocytes incubated with TBBPS at 1 to 100 μg/mL for 48 h.

**Figure 17:**
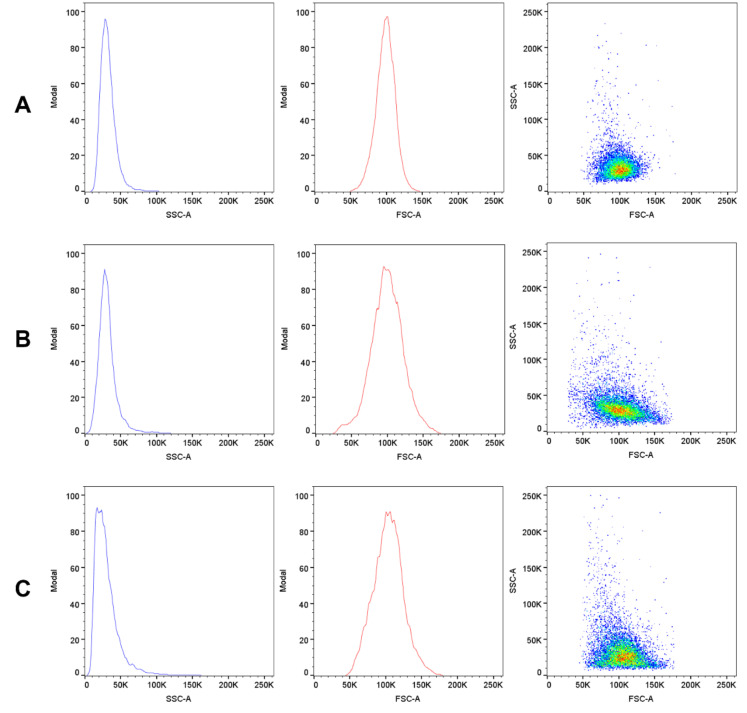
Scattering diagrams of human control erythrocytes (**A**), erythrocytes incubated with TBBPS at 100 µg/mL (**B**) and erythrocytes incubated with TBBPA at 25 µg/mL (**C**) for 48 h. The FSC-A diagrams represent the light scattered near the forward direction (proportional to the value of the particles). The SSC-A diagrams represent scattering at a right angle (depended on cell shape and internal properties). The FSC-A/SSC-A diagram is a dual parameter contour plot proportional to the total cell diversity.

In the case of SSC, it was found that TBBPA caused a slight statistically significant increase of the parameter at concentrations of 10 and 15 µg/mL (103, 104%, respectively), while at the concentration of 25 µg/mL the compound caused a significant decrease in relation to the control (71%) (Figure 15). In the case of TBBPS, no statistically significant changes were found (Figure 16) within the range of presented concentrations. The histograms and SSC/FSC dot plots of control and TBBPA and TBBPS in the final concentration were found in Figure 17.

### 2.9. Microscopic Analysis

**Figure 18:**
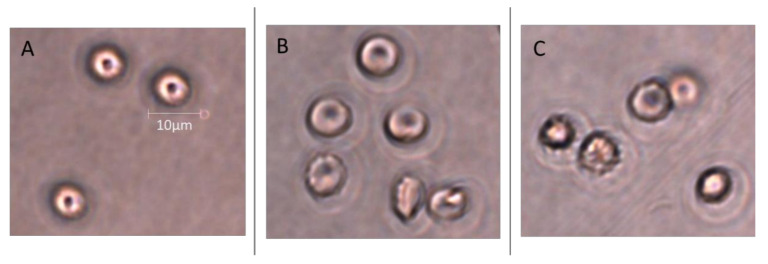
Micrographs of human control erythrocytes (**A**) and the erythrocytes incubated with TBBPA at 25 μg/mL (**B**) and TBBPS at 100 μg/mL (**C**) for 48 h.

Microscopic analysis confirmed that 48 h incubation of TBBPA and TBBPS induced morphological changes within cells. Pictures were taken for TBBPA at concentrations of 25 µg/mL, and for TBBPS at concentrations of 100 µg/mL. The discussed compounds induced the formation of echinocytes (Figure 18).

## 3. Discussion

The protein and lipid components of the RBC membrane create a complex structure of interrelationships enabling the maintenance of the proper shape and physiology. Disturbances in these membrane components may cause changes in the shape and deformation capacity of erythrocytes, which may contribute to disturbances in their function and shorten their lifespan [19]. In this study, we assessed the effects of TBBPA and TBBPS on the erythrocyte membrane, which is the first barrier to xenobiotics entering circulation.

One of the properties of a biological membrane, resulting from its structure and interactions between its components, is fluidity [20]. Using the electron paramagnetic resonance (EPR) method, the placement of 5-DSA and 16-DSA probes in the erythrocyte membrane exposed to TBBPA and TBBPS was assessed. These probes diffuse into the environment of the lipid bilayer of biological membranes: 5-DSA is located with the hydrocarbon chains, and 16-DSA is located deeper, in the middle of the lipid bilayer. Under the influence of TBBPA or TBBPS, a statistically significant increase in fluidity of the hydrophilic layer of the RBC membrane was observed (as evidenced by an increased order parameter S). An upward trend was also observed in the deeper regions of the lipid bilayer, but it was statistically insignificant (Table 1). It can be assumed that the analyzed compounds, due to the presence of bromine atoms in them (large in size), initially localized in the shallower regions of the lipid bilayer. The inverse ability to locate in the membrane was demonstrated by Maćczak et al. (2017) in their studies on the effects of bisphenol A and its analogues on RBCs [21]. These authors found that bisphenols did not affect the S parameter, and therefore, did not localize in the hydrophilic layer, but changed the relaxation times τB and τC, which indicates penetration of compounds into the hydrophobic layer, which is explained by the high hydrophobicity of the analyzed compounds [21]. Perhaps the additional presence of bromine atoms in these compounds makes it difficult for them to penetrate deeper regions to the level of carbon 16, where bisphenols devoid of bromine atoms were located.

The W/S parameter determines the state of the internal conformation of membrane proteins, so it is a very sensitive parameter determining changes in their properties. This parameter was also tested with the use of the EPR method by using the MSL spin marker covalently binding to the sulfhydryl groups (-SH) of cytoskeleton proteins, mainly spectrin and actin. It was found that TBBPA, starting from the concentration of 1 µg/mL, caused a decrease in the mobility of the attached marker, and TBBPS caused an increase starting from the concentration of 50 µg/mL (Figure 1 and Figure 2). An increase in the W/S ratio may indicate conformational changes in the structures of membrane proteins that lead to higher exposure of thiol groups to chemical reactions, and/or may contribute to disulfide bond breakage. On the other hand, a decreased W/S ratio may indicate oxidation of thiol groups by reactive oxygen species (ROS), which reduces their availability for the marker. The increase in ROS level under the influence of TBBPA, even at very low concentrations (0.001 µg/mL), was confirmed in another paper [22]. A decrease in W/S may also indicate formation of protein aggregates, which may also be caused by ROS [23,24]. Moreover, we found that the analyzed compounds contributed to increases in the level of thiol groups (TBBPA from 25 µg/mL, and TBBPS from 50 µg/mL) (Figure 3 and Figure 4), which may partially confirm the influences of TBBPA and TBBPS on the conformation of membrane proteins caused by interactions with thiol groups. Pocernich et al. (2001) also found that lipid peroxidation products could covalently bind to cysteine, lysine or histidine, which may also result in conformational changes of membrane proteins [25]. In the case of our research, a statistically significant increase in lipid peroxidation was found only after incubation of RBCs with TBBPS at the highest concentration (Figure 8). Moreover, in the previous paper [26] we found that TBBPA (at the concentration of 25 µg/mL) and TBBPS (at the concentration of 250 µg/mL) caused decreases in tryptophan fluorescence in erythrocyte membranes, which also confirms that these compounds may damage membrane proteins via ROS.

Changes in the viscosity of the interior of RBCs may be associated with changes in their shape. Although we observed upward trends in the assessment of intrinsic viscosity of erythrocytes, these changes were not statistically significant (Table 2). On the other hand, it was found that the analyzed compounds induced changes in the forward scatter channel (FSC) and side scatter channel (SSC) parameters obtained from flow cytometric analysis (Figure 13, Figure 14, Figure 15, Figure 16 and Figure 17), which may reflect changes in the size, shape and external structure of the membranes of the investigated cells. These changes are also visible in the pictures achieved from a phase-contrast microscopic examination (Figure 18). An increase in the size of erythrocytes and changes in their shape are usually associated with damage done to the cell membrane, and may result from influx of water into the cell and/or incorporation of compounds into the structure of the membrane. It is also known that cytoskeleton proteins and integral membrane proteins are responsible for maintaining the shape of RBCs. Various xenobiotics, including phenols, can damage erythrocyte proteins [27,28], resulting in conversion of a normal discocyte into an echinocyte or a stomatocyte [29]. In the case of compounds analyzed by us, formation of echinocytes may be conditioned by incorporation of TBBPA or TBBPS into the hydrophilic region of the membrane, as indicated by changes in the S parameter. A decrease in the ATP level in the cell may also contribute to changes in the shape of the cell, and thus to a decrease in its survival [30]. We observed that both TBBPA and TBBPS significantly decreased cellular ATP levels—the highest concentrations by nearly 60% with TBBPA (25 µg/mL), and more than 40% when incubated with TBBPS at 100 µg/mL.

Shape changes can also be associated with water loss, increased intracellular viscosity and decreased osmotic resistance in RBCs. In the case of our research, slight increases in osmotic resistance were observed when RBCs were incubated with TBBPA at concentrations of 15 and 25 µg/mL (Figure 9), which could also have been related to incorporation of the compound into the membrane and its partial stiffening, which would result in reduced susceptibility to hemolysis [29,31]. Moreover, in the case of this compound, the IC_50_ value decreased as the concentration increased, which may confirm the above observations.

## 4. Materials and Methods

### 4.1. Chemicals

TBBPA (purity 99%, 2,6-dibromo-4-[2-(3,5-dibromo-4-hydroxyphenyl)propan-2-yl]phenol)) was purchased from LGC Standards (Wesel, Germany). Tetrabromobisphenol S (purity 98.8%, 2,6-dibromo-4-(3,5-dibromo-4-hydroxyphenyl)sulfonylphenol) was synthetized in the Institute of Industrial Organic Chemistry in Warsaw, Poland. DMSO (99.5%), 16-doxylstearic acid (16-DSA), 4-N-maleimide-2,2,6,6-tetramethylopiperidine-1-oxyl (MSL), 2,2,6,6-tetramethyl piperidine-Noxyl-4-amine (TEMPAMINE) and ouabain were bought from Sigma-Aldrich (Merck, Kenilworth, NJ, USA). 5-Doxylstearic acid (5-DSA) was bought from Santa Cruz Biotechnology (Dallas, TX, USA). ATP Determination Kit was purchased from Thermo Fisher Scientific (Waltham, MA, USA). Ethylenediaminetetraacetic acid tetrasodium salt (EDTA), tris (hydroxymethyl)aminomethane (Tris), 5,5-dithiobis-2-nitrobenzoic acid (DTNB), sodium dodecyl sulfate (SDS), phenylmethylsulfonyl fluoride (PMSF) and other chemicals were obtained from Carl Roth (Roth, Germany), POCh, (Gliwice, Poland) or Alfachem (Lublin, Poland).

### 4.2. Erythrocyte and Erythrocyte’s Membranes Isolation

RBCs were isolated from leukocyte-buffy coat separated from blood from healthy donors from the Regional Centre of Blood Donation and Blood Treatment (Lodz, Poland).

The RBCs’ isolation and treatment procedure was previously described by Jarosiewicz et al. (2017) [32]. RBCs with a hematocrit of 5% (about 630 mln cells x mL^−1^) were incubated with the analyzed compounds at concentrations ranging from 1 to 25 µg/mL for TBBPA and 1–250 µg/mL for TBBPS, at 37 °C for 48, 12 or 3 h, depending on the experiment. Differences in the concentrations of the compounds studied were dictated by hemolytic properties of BFRs tested. Compounds were dissolved in DMSO (to final concentration of 0.4%). Concentrations of the compounds were selected on the basis of their hemolytic abilities described in the previous paper [32]. Moreover, in the case of the 48 h incubation period, an additional antibiotic was used (0.2% streptomycin and penicillin). Appropriate controls were performed to exclude the effect of antibiotic and DMSO on RBCs. The exact conditions of incubation are described in the article by Jarosiewicz et al., 2020 [26].

Isolation of RBCs membranes followed the incubation of the RBCs with the analyzed compounds. Isolation of RBC membranes was carried out using the Dodge et al. (1963) method with some modifications [33]. The exact isolation procedure was described in the previous paper by Jarosiewicz et al., 2020 [26].

The research was approved by the Bioethics Committee of the University of Lodz No. 7/KBBN-UŁ/II/2015.

### 4.3. Membrane Fluidity

The RBC’s membrane fluidity was analyzed by electron paramagnetic resonance (EPR) spectroscopy (Brucker 300 Spectrometer, Ettlingen, Germany) using spin labeled fatty acids: 5-doxylstearic acid (5-DSA) and 16-doxylstearic acid (16-DSA). From the EPR spectra obtained for the 5-DSA spin label, the ordering parameter S was calculated, and the correlation times τB and τC were calculated for the 16-DSA spin label. Order parameter S and the correlation times τB and τC were calculated as described in the study of Koter et al. (2004) [34].

### 4.4. W/S Ratio

Parameter W/S was determined using a spin label MSL, which covalently binds proteins and analyzed by EPR spectroscopy (Brucker 300 Spectrometer, Ettlingen, Germany). The exact procedure for performing the experiment is described in the article by Maćczak et al. (2017) [21].

### 4.5. Internal Viscosity

The TEMPAMINE spin label was used to determine the intracellular environment of RBCs [35]. The analysis was conducted using Brucker 300 Spectrometer (Ettlingen, Germany). The changes in the parameter studied were calculated and expressed as percentages of control. The exact procedure for performing the experiment is described in the article by Maćczak et al. (2017) [21].

### 4.6. Thiol Groups Level

The number of thiol groups in the erythrocyte membranes was determined using the method of Ellman et al. (1959) [36]. 5.5′-Dithiobis (2-nitrobenzoic) acid reacts with protein thiol groups. This reaction releases the 5-thio-2-nitrobenzoic anion having an intense yellow color, which is determined spectrophotometrically at 412 nm wavelength. The procedure of determination of the thiol group level was previously described by Maćczak et al. (2017) [21]. Results are expressed as -SH nmol/mg proteins and presented as percentages of control.

### 4.7. ATP Level

Intracellular ATP level in RBCs is determined by oxidative decarboxylation of luciferin by firefly luciferase in the presence of ATP and magnesium ions with bioluminescence emission. The emission is linearly related to the intracellular ATP concentration [37]. The measurements were made at the wavelength of 590 nm using fluorimeter (Fluoroskan Ascent FL, Thermo Fisher Scientific, Vantaa, Finland). The exact procedure for performing the experiment is described in an article by Maćczak et al. (2017) [21].

### 4.8. Lipid Peroxidation

Lipid peroxidation in erythrocyte membranes is determined according to the method of Stocks and Dormandy (1971) [38]. Lipid peroxidation is analyzed by measuring of formation of thiobarbituric acid reactive substances (TBARS). The absorbance is determined colorimetrically using BioTek ELx808 reader (Winooski VT, USA) at the wavelength of 532 nm. Lipid peroxidation is expressed in absorbance units of TBARS products and is shown as a percentage of control.

### 4.9. Osmotic Fragility

The osmotic resistance (fragility) was determined by the method of Dacia and Lewis (1975) [39]. A small number of erythrocytes are placed in a NaCl solution at a concentration of 0.2 to 0.9%. Osmotic resistance is determined by measuring the hemoglobin released from erythrocytes by the colorimetric method using the BioTek ELx808 reader (Winooski VT, USA) at λ = 540 nm. Osmotic resistance is assessed on the basis of the hemolysis curve shift, in the graph of percentage of hemolysis vs. NaCl concentration. Before performing the assay, RBCs were incubated with test compounds for 3 h. The exact procedure for performing the experiment is described in the publication by Maćczak et al. (2017) [21].

### 4.10. Morphological Changes of Erythrocytes (FSC and SSC Parameter)

The flow cytometry technique was used to assess the size and shape of the erythrocytes (LSR II Becton Dickinson). Data were recorded for a total of 10,000 events per sample. Results are presented as percentages of control. This method was described by Bukowska et al. (2011) [28].

### 4.11. Microscopic Analysis

Microscopic analysis was completed using the phase contrast microscope (Olympus, Japan) at the magnification of 600×. Images were taken following a 48 h incubation of RBCs with analyzed compounds. After incubation, RBCs were suspended in Ringer’s buffer at the final concentration of 0.02%, placed on a Petri dish and pictures were taken.

### 4.12. Statistical Analysis

Results are presented as means ± standard deviations of 4–6 experiments (blood donors); each experiment performed was the mean of 2–3 replicates. The statistical analysis was described in the previous article by Jarosiewicz et al. (2020) [26].

## 5. Conclusions

Both TBBPA and TBBPS were found to cause changes in the erythrocyte cell membrane. Both compounds increase the fluidity of the hydrophilic region of the RBC membrane. TBBPA strongly damages proteins (changes in the S, W/S ratio, level of thiol groups, and levels of tryptophan oxidation in membranes and in human albumin, as shown in previous studies) [26]. In our opinion it is the main target of this retardant. It was also shown that TBBPS contributed to lipid peroxidation only at its highest concentration of 250 µg/mL, which may indicate that the peroxidation process will be a secondary process to the induction of ROS and protein oxidation by these compounds [22]. Both compounds also caused changes in the shape and size of erythrocytes, which are associated with damage to the cell membrane, hemolysis and incorporation of these compounds into the structure of the membrane. In addition, the induced decrease in the level of ATP would contribute to a decrease in cell survival. It is worth noting that changes in the structure and function of the cell membrane were observed for significantly lower concentrations in the case of RBC incubation with TBBPA than with TBBPS, occurring only at occupational and not epidemiological exposure. The obtained data indicate a low toxicity of TBBPS only at very high concentrations (in contrast to TBBPA), and therefore, a low toxicological risk posed by this retardant to human erythrocytes.

## Figures and Tables

**Table 1:** Changes in parameter S and correlation times of τB and τC in human control erythrocytes and the erythrocytes incubated with TBBPA at 1 to 25 μg/mL and TBBPS at 1 to 100 μg/mL for 48 h. (*) Significantly different from control (*p* < 0.05).

Compound	Concentration [µg/mL]	Order Parameter S [%]	Correlation Time τ_B_ [%]	Correlation Time τ_C_ [%]
TBBPA	0	100.00 ± 0.009	100.00 ± 0.444	100.00 ± 0.476
	1	101.86 ± 0.003 *	103.75 ± 0.691	104.92 ± 0.925
	10	103.01 ± 0.002 *	104.79 ± 0.823	103.95 ± 1.021
	15	103.95 ± 0.001 *	105.24 ± 0.727	103.70 ± 1.387
	25	105.45 ± 0.009 *	105.05 ± 0.740	104.32 ± 0.887
TBBPS	0	100.00 ± 0.009	100.00 ± 1.080	100.00 ± 1.305
	1	102.57 ± 0.010	100.47 ± 0.231	102.37 ± 0.925
	10	104.45 ± 0.009 *	100.27 ± 0.433	101.32 ± 0.774
	50	105.99 ± 0.008 *	104.18 ± 0.735	104.40 ± 0.455
	100	108.33 ± 0.021 *	106.93 ± 0.903	108.38 ± 1.330

**Table 2:** Internal viscosity of human control erythrocytes and the erythrocytes incubated with TBBPA at 1 to 25 μg/mL incubated and TBBPS at 1 to 100 μg/mL for 48 h. (*) Significantly different from control (*p* < 0.05).

Compound	Concentration [µg/mL]	Internal Viscosity [%]
TBBPA	0	100.00 ± 0.004
	1	103.32 ± 0.004
	10	104.22 ± 0.005
	15	105.19 ± 0.004
	25	106.40 ± 0.004
TBBPS	0	100.00 ± 0.003
	1	99.42 ± 0.004
	10	99.53 ± 0.007
	50	103.37 ± 0.006
	100	107.50 ± 0.007

## Abbreviations

BFRs	brominated flame retardants
TBBPA	tetrabromobisphenol A
TBBPS	tetrabromobisphenol S
ATP	adenosine triphosphate
RBCs	erythrocytes
FSC	forward scatter channel
SSC	side scatter channel
EPR	electron paramagnetic resonance
ROS	reactive oxygen species
